# Neurobiological effects of microbial treatments within psychiatry: a systematic review

**DOI:** 10.3389/fpsyt.2026.1745964

**Published:** 2026-04-20

**Authors:** Cassandra Sgarbossa, Evan Forth, Scott Squires, Ashley Groth, Maria Farid, Katherine Gallant, Dharmayu Desai, William Redfearn, Roumen Milev

**Affiliations:** 1 Centre for Neuroscience Studies, Queen’s University, Kingston, ON, Canada; 2 Providence Care Hospital, Kingston, ON, Canada; 3 Department of Psychiatry, Queen’s University, Kingston, ON, Canada; 4 Department of Psychology, Queen’s University, Kingston, ON, Canada

**Keywords:** gut microbiome, gut-brain axis, imaging, probiotics, psychiatry

## Abstract

**Objective:**

Though microbial interventions such as probiotics and fecal microbiota transplantation have had a growing body of evidence suggesting their efficacy in alleviating the symptoms of psychiatric illnesses, their exact mechanisms of action and impacts on the brain are still not fully characterized. The aim of this review is to compile and summarize the current literature regarding neurobiological changes associated with microbial interventions targeting psychiatric symptoms in healthy and psychiatric populations.

**Methods:**

A systematic search of four databases was conducted using key terms related to neuroimaging, microbial interventions, and psychiatric illnesses and/or symptoms. All results were then evaluated based on specific eligibility criteria.

**Results:**

10 studies met eligibility criteria and were included in this systematic review. Three of the five healthy control studies and all five of the studies conducted within psychiatric populations, observed significant neurobiological changes associated with probiotic intervention either in areas with psychiatric relevance, in the direction of a healthier profile, or correlated with improved psychiatric and/or affective symptoms. The interventions used in these studies consisted of probiotics with bacterial species primarily from the *lactobacillus* and *bifidobacterium* genera, at doses ranging from 1–900 billion CFU, taken for durations ranging from 4 weeks to 6 months.

**Conclusions:**

The findings from this review suggest that probiotic intervention may be associated with neurobiological changes, and that these changes could play a role in ameliorating psychiatric symptoms. More research is needed to replicate these findings, explore other psychiatric populations and microbial interventions, and fully elucidate the mechanisms driving these promising neurobiological and clinical changes.

## 1 Introduction

While there have been a variety of treatment options that have existed throughout history as interventions for psychiatric disorders, common current treatments often include pharmacotherapy (e.g., antidepressant medications, mood stabilizers, antipsychotics, etc.) and/or psychotherapy (e.g., cognitive behavioral therapy) (1, 2). However, given the heterogenous nature of psychiatric disorders and unique variability between individuals, approximately 20 to 60% of patients can still experience treatment resistance (3). In efforts to optimize patient care and better understand the potential mechanisms involved with treatment response, there has been a push to explore novel and unconventional treatment options. Recently, microbial interventions targeting the gut microbiome (e.g., probiotics, fecal microbiota transplant [FMT], etc.) have gained traction and interest as potential target for treating psychiatric disorders (4, 5).

The gut microbiome is a complex ecosystem of living bacteria that exist within the human gut. In fact, the gut is home to approximately 100 trillion microbes, which is ten times larger in quantity than there are cells in the human body (6). While gut microbiome development is primarily critical during the first three years of life, it is continuously modified throughout life based on external influences such as geographical location, stress, medication use, and/or diet (7). Furthermore, despite Bacteroidetes and Firmicutes being the two predominant bacterial phyla found in healthy adult humans, external influences can still alter microbial composition over time and play a role in the development of dysbiosis (8). This becomes evident in clinical settings, as certain psychiatric disorders such as major depressive disorder (MDD), have been associated with systemic inflammation (9, 10), gut dysbiosis (11, 12), and altered gut microbiome composition (13–15).

The dynamic ability of the gut microbiome to influence psychological states and overall mood is suggested to be mediated by the Gut-Brain Axis (GBA), a bidirectional signaling pathway between the gastrointestinal (GI) tract and central nervous system (4). The GBA is essential in regulating various physiological and homeostatic processes within the body (16), while also aiding in the integration of communication from the nervous, immune, and GI systems (17). These processes by which the gut microbiome is able to modulate mood via the GBA, is still unclear. The nature of these processes is not mutually exclusive – but rather, they are an entanglement of systems interacting with one another. They interact with both direct and indirect pathways, such as endocrine (e.g., hypothalamic-pituitary-adrenal (HPA) axis regulation) (18, 19), neural (e.g., vagus nerve interactions) (20–22), and immune pathways (e.g., involvement of inflammatory cytokines) (10, 23, 24).

Microbial interventions have long been used to treat various GI-related diseases, such as irritable bowel disease (IBD), clostridium difficile (CD) (25), and ulcerative colitis (UC) (26), with the purpose of repopulating and/or altering the gut microbiome through microbiota manipulation. These interventions often include but are not limited to: various bacteriotherapeutic options such as prebiotics, probiotics, and synbiotics (27, 28), FMT (26), and novel FMT alternatives (29). FMT, a well-documented treatment method that aims to restore microbial balance of the gut microbiome, involves the transfer of fecal matter from a healthy donor to the intestinal tract of an unwell recipient via colonoscopy. While traditionally used as treatment for CD infections (30) and IBD (31, 32), it has recently been investigated for its use in treating psychiatric disorders (29, 33, 34), however more work is needed to confirm these findings and better understand FMT’s role as a psychiatric therapeutic agent.

In addition to FMT, the use of probiotic supplementation for the management of psychiatric symptoms and mood has also been explored, due to its increased feasibility and cost-effective nature in comparison to other microbial interventions (35–37). Probiotics work by introducing live bacteria to the gut microbiome in efforts to maintain its health and functioning (17, 35). L*actobacillus* and *Bifidobacterium* are amongst the most common genus found in probiotic supplementation targeted for use within psychiatry, due to their suggested influence on clinical symptoms and outcomes (38–40). As a treatment for depression, probiotics have been associated with improvements in cognitive performance, reduced neurodegenerative compounds (e.g., kynurenine) (41), improved depressive symptomology (39), and improved memory recall (42).

Neuroimaging use within psychiatry has been used to identify structural and functional characteristics associated with specific disorders and better understand any involved neural mechanisms (43). Structural techniques, such as computerized tomography (CT), voxel-based morphometry (VBM), magnetic resonance imaging (MRI), and diffusion tensor imaging (DTI) provides information on brain structure and size, visualization of grey and white matter composition, and gross brain abnormalities such as tumors (44). In contrast, functional techniques, such as functional magnetic resonance imaging (fMRI), positron emission tomography (PET), and electroencephalography (EEG), can measure the activity levels of different parts of the brain, which researchers and clinicians can associate with cognitive/behavioral tasks and various clinical predictors and outcome variables to guide research and treatment (44–46).

Recently, there has been a surge of neuroimaging use within psychiatry to help identify distinct pathophysiological mechanisms underlying various psychiatric disorders and treatments, but this area of research in relation to the gut microbiome is still in its nascency. Though some systematic reviews and meta-analyses have been conducted with the focus of elucidating the efficacy of probiotics in alleviating the symptoms of specific psychiatric illnesses, there has yet to be a review on the neurobiological findings associated with gut microbiome manipulation. Due to the novel nature of this research, the aim of this review is to further explore any neural and/or neurobiological changes associated with microbial interventions, within the scope of a psychiatric setting. This research serves to better understand the effect of microbial changes throughout the body, with massive potential and implications for optimizing patient health of individuals experiencing a psychiatric illness or mood disturbance.

## 2 Methods

### 2.1 Literature search strategy

**Figure 1:**
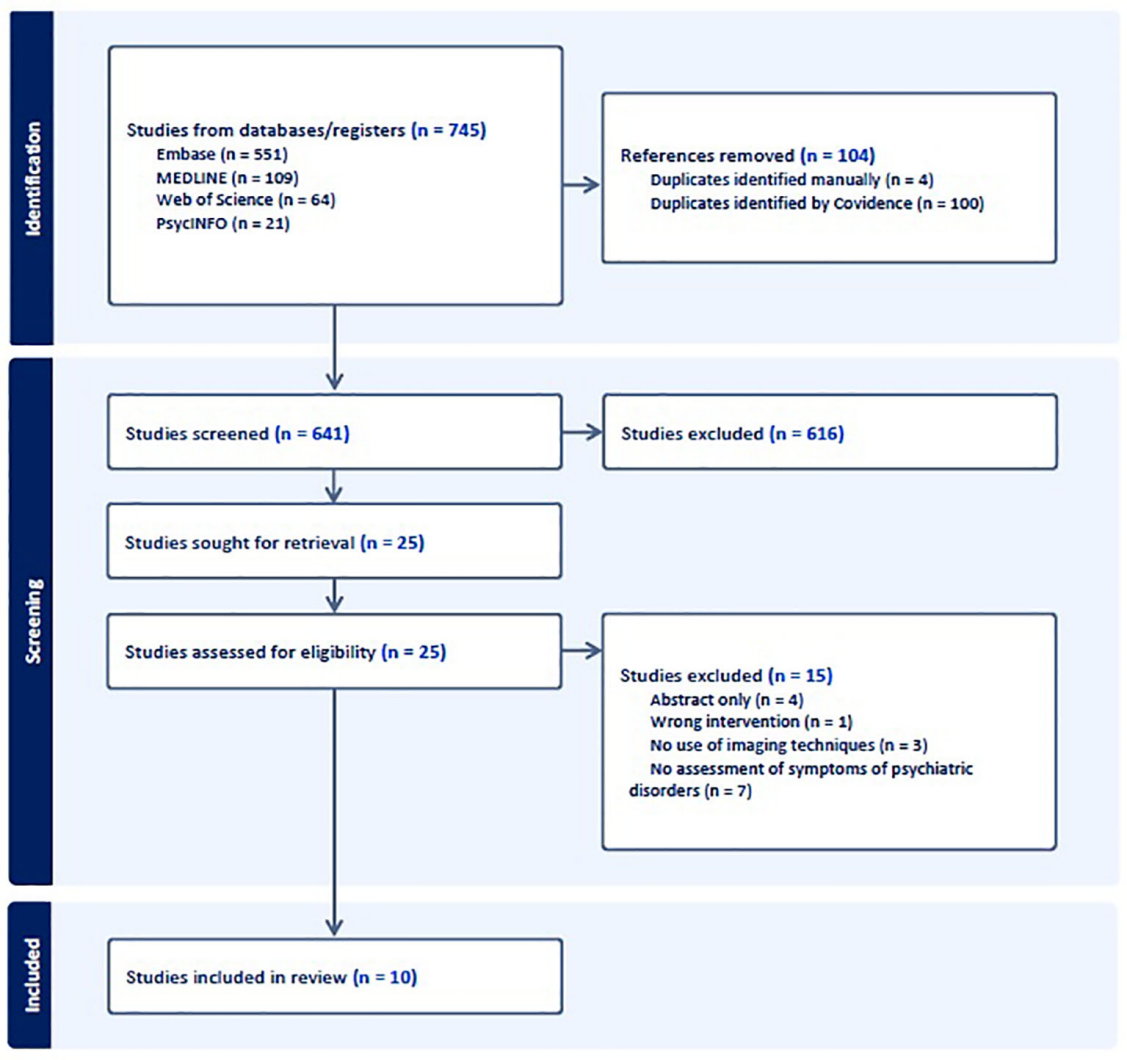
PRISMA flow chart detailing the identification and selection of studies for review.

This review followed the Preferred Reporting Items for Systematic Reviews and Meta-Analyses (PRISMA) guidelines for systematic reviews in unison with Covidence, a primary screening and data extraction tool (47). A total of four databases (MEDLINE, PsycINFO, EMBASE, and Web of Science) were utilized in the search strategy, including all relevant articles from inception to date of search on January 15^th^, 2025. Key search terms were developed with the intention of identifying any studies that utilized neuroimaging tools, used a microbial intervention, and collected psychiatric symptom data. Studies that collected psychiatric symptoms in a healthy population, or psychiatric symptoms secondary to physical illnesses, were included in addition to studies in psychiatric populations. Limiting studies to the assessment of psychiatric symptoms ensured that microbial interventions were designed to primarily affect psychiatric symptoms, rather than physical symptoms. The terms used through the Ovid interface for MEDLINE, PsycINFO, and EMBASE databases included: (magnetic resonance imaging OR MRI OR functional magnetic resonance imaging OR fMRI OR diffusion tensor imaging OR DTI OR positron emission tomography OR electroencephalography OR EEG OR computerized tomography OR CT OR functional near-infrared spectroscopy OR fNIRS OR ultrasound OR magnetoencephalography) AND (probiotic* OR prebiotic* OR postbiotic* OR psychobiotic* OR fecal microbiota transplant* OR FMT OR stool transplant OR bacteriotherapy OR microbe therapy OR microbe transfer) AND (depress* OR MDD OR bipolar* OR attention deficit hyperactivity disorder OR ADHD* OR autism* OR ASD OR anxiety* OR mania OR schizo* OR obsessive compulsive disorder OR OCD OR posttraumatic stress disorder OR PTSD). The database-specific search strategy and query syntax for search terms can be found in the **Supplementary File 1**. The search yielded 745 studies after 104 duplicates were removed. After abstract screening, 25 studies were assessed for eligibility through full-text review and 15 were excluded for either: no assessment of psychiatric symptoms, conference abstracts and/or abstract only, no use of imaging, or wrong intervention. The remaining 10 studies passed all criteria and were included in the review. For a detailed overview of the screening process, please see **Figure 1**.

### 2.2 Eligibility criteria

The following inclusion criteria were required to be eligible for the review: (1) Must utilize a neuroimaging technique either a) during intervention; b) after intervention; or c) during and after intervention; (2) Must assess for symptoms of psychiatric disorders; (3) Subjects must have undergone a form of microbial-based intervention; (4) Studies written in English and published in a peer-reviewed journal. No limits were placed on study design as long as the other inclusion criteria was met. No other criteria were used to exclude studies from review.

### 2.3 Study selection

Author C.S. completed all literature searches. Authors W.R. and D.D completed the abstract screening, M.F. and K.G. completed full-text review, and any conflicts between reviewers was resolved by a third party, C.S. Author A.G. conducted data extraction systematically using the data fields listed in **Table 1**. Extracted data was then reviewed and confirmed by S.S. E.F. completed the study quality assessment shown in **Table 2**, **3**.

**TABLE 1:** Study Characteristics.

Study	Sample			Primary Indication	Study Design	Intervention	Timeline	Imaging Measures	Clinical Measures	Results
	Sample Size	Age (Mean)	Sex							
Healthy Indication										
Rode (48)	*n*=22	24.2	16F, 6M	Healthy controls	DBRCT	Probiotic (containing: Bifidobacterium longum, Lactobacillus helveticus, and Lactiplantibacillus plantarum) (3x10^9^ CFU) *vs.* Placebo	4 weeks of treatment, 4 weeks of washout, then 4 weeks of alternate treatment.	MRI, VBM	EQ-5D-5L, HADS, PSS, STAI, KSD, DOW	Probiotic intervention resulted in significant functional connectivity changes in the default mode, salience, and frontoparietal networks, as compared to placebo. Psychological symptoms improved non-significantly, after probiotic intervention.
Ascone (49)	*n*=59	27.1	33F, 26M	Healthy controls	DBRCT	Probiotic (containing: Lactobacillus paracasei, Lactobacillus plantarum, Lactobacillus acidophilus, Lactobacillus helveticus, Bifidobacterium lactis, Bifidobacterium breve, and Streptococcus thermophilus) (450x10^9^ CFU) *vs.* Placebo	4 weeks of treatment	MRI	BDI-II-R, BSI, PSS, RSQ	No significant changes in hippocampal grey matter and functional connectivity. No significant group x time interactions for whole-brain grey or white matter. No significant changes in psychiatric symptoms.
Allen (50)	*n*=22	25.5	All males	Healthy males	Repeated measures, placebo-controlled study	Probiotic (containing: Bifidobacterium longum) (CFU not reported) *vs.* Placebo	4 weeks of placebo, followed by 4 weeks of probiotics treatment.	EEG	CPSS	Daily stress levels were lower during probiotic intervention but returned to elevated levels during the 2-week follow-up. For EEG, Fz mobility differed significantly across conditions and was significantly higher post-probiotic treatment. Cz theta power was significantly lower post-probiotic treatment, as compared with post-placebo.
Kelly (51)	*n*=29	24.6	All males	Healthy males	Randomized Crossover Trial	Probiotic (containing: Lactobacillus rhamnosus) (1x10^9^ CFU) *vs.* Placebo	8 weeks of treatment	EEG	BDI, PSQI, PSS, SAI, TAI, SCL-90	No significant differences between probiotic and placebo group.
Papalini (52)	*n*=58	21.5	All females	Healthy females	DBRCT	Probiotic (containing: Bifidobacterium bifidum, Bifidobacterium lactis, Bifidobacterium lactis, Lactobacillus acidophilus, Lactobacillus brevis, Lactobacillus casei, Lactobacillus salivarius, and Lactococcus lactis) (5x10^9^ CFU) *vs.* Placebo	4 weeks of treatment	fMRI	BDI, LEIDS-R, Emotional face-word Stroop paradigm, Color-word Stroop paradigm	With stress induction, the probiotic group demonstrated a significant increase in working memory performance after supplementation, as compared to placebo.
Psychiatric Indication										
Billeci (53)	*n*=46	46.5 months	11F, 35M	ASD	DBRCT	Probiotic (containing: Streptococcus thermophilus, Bifidobacterium breve, Bifidobacterium longum, Bifidobacterium infantis, Lactobacillus acidophilus, Lactobacillus plantarum, Lactobacillus paracasei, and Lactobacillus delbrueckii subsp. Bulgaricus) (CFU not reported) *vs.* Placebo	6 months of treatment	EEG	GSI, ADOS-2 CARS, SCQ, RBS-R, CBCL, GMDS-R, VABS-II, CDI	In the probiotic group, there was a decrease of power in frontopolar regions in beta and gamma bands and increased coherence in beta and gamma bands with a shift in frontal asymmetry. EEG measures were significantly correlated with clinical measures, as there was a significant association between frontopolar coherence in the beta and gamma bands and the "writing skills" domain of the VABS-II.
Schaub (39)	*n*=47*	39.1	27F, 20M	MDD	DBRCT	Probiotic (containing: Streptococcus thermophilus, Bifidobacterium breve, Bifidobacterium longum, Bifidobacterium infantis, Lactobacillus acidophilus, Lactobacillus plantarum, Lactobacillus paracasei, Lactobacillus delbrueckii subsp. Bulgaricus) (900x10^9^ CFU) *vs.* Placebo	31 days of treatment	fMRI, VBM	HAM-D	There was a significant decrease in HAM-D scores over time, with a stronger effect in the probiotic group in comparison to the placebo group. No significant effects in grey matter volume, however the probiotics group demonstrated increased grey matter volume in the calcarine sulcus after intervention, in comparison to placebo. Putamen activation in response to neutral faces had a significant decrease after probiotic intervention, in comparison to placebo.
Schneider (42)	*n*=43*	38.6	26F, 17M	MDD	DBRCT	Probiotic (containing: Streptococcus thermophilus, Bifidobacterium breve, Bifidobacterium longum, Bifidobacterium infantis, Lactobacillus acidophilus, Lactobacillus plantarum, Lactobacillus paracasei, Lactobacillus delbrueckii subsp. Bulgaricus) (900x10^9^ CFU) *vs.* Placebo	31 days of treatment	fMRI	VLMT, Corsi Block Tapping Test, Trail Making Test	Immediate recall in the VLMT significantly improved after probiotic intervention. There was also a significant difference in hippocampus activation during working memory processing in the probiotic group compared to placebo. Other measures did not reveal significant changes.
Yamanbaeva (54)	*n*=32*	37.6	18F, 14M	MDD	DBRCT	Probiotic (containing Streptococcus thermophilus, Bifidobacterium breve, Bifidobacterium longum, Bifidobacterium infantis, Lactobacillus acidophilus, Lactobacillus plantarum, Lactobacillus para-casei, Lactobacillus delbrueckii subsp. Bulgaricus) (900x10^9^ CFU) *vs.* Placebo	31 days of treatment	fMRI	HAM-D	Probiotics maintained mean diffusivity in the left uncinate fasciculus and altered rsFC between limbic structures and the precuneus. A cluster in the left superior parietal lobule showed altered rsFC to the subcallosal cortex, left orbitofrontal cortex, and limbic structures after probiotic intervention. Decreases in rsFC from the right amygdala to the cluster in the superior parietal lobule were related to decreased in depressive symptoms.
Pinto-Sanchez (55)	*n*=44	43.25 (median)	24F, 20M	IBS	DBRCT	Probiotic (containing: Bifidobacterium longum) (10x10^9^ CFU) *vs.* Placebo	6 weeks of treatment	fMRI	HAD, STAI, Birmingham IBS Score, Bristol stool scale, self-reported IBS improvement, SF-36, PHQ-15	At week 6, 64% of probiotic participants and 32% of placebo participants had a reduction in HAD scores. Probiotics had no significant effects on anxiety or IBS symptoms. Participants in the probiotic group had a mean increase in QoL scores as determined by the SF-36, as compared to the placebo group. The probiotic group also displayed reduced activity in response to negative emotional stimuli in the amygdala and fronto-limbic regions, as compared to placebo.

ADOS-2: Autism Diagnostic Observation Schedule – Second Version; ASD: Autism Spectrum Disorder; BDI: Beck Depression Inventory; BDI-II-R: Beck Depression Inventory (2e) – Revised; BSI: Brief Symptom Inventory; CARS: Childhood Autism Rating Scale; CBCL: Child Behavior Checklist; CDI: McArthur-Bates Communicative Development Inventories; CFU: Colony Forming Unit; CPSS: Cohen Perceived Stress Scale; Cz: Midline Central; DBRCT: Double-Blind Randomized Controlled Trial; DOW: Diary of Workload; EEG: Electroencephalography; EQ-5D-5L: EuroQol 5-Dimension 5-Level Health-Related Quality of Life Index; fMRI: functional Magnetic Resonance Imaging; Fz: Midline Frontal; GMDS-R: Griffiths Mental Development Scales - Extended Revised; GSI: Gastrointestinal Severity Index; HAD: Hospital Anxiety and Depression Scale; HAM-D: Hamilton Depression Rating Scale; IBS: Irritable Bowel Syndrome; KSD: Karolinska Sleep Diary; LEIDS-R: Leiden Index of Depression Sensitivity - Revised; MRI: Magnetic Resonance Imaging; PHQ-15: Patient Health Questionnaire-15; PSS: Perceived Stress Scale; QoL: Quality of Life; RBS-R: Repetitive Behavior Scale-Revised; RCT: Randomized Controlled Trial; RSQ: Response Styles Questionnaire; SAI: State Anxiety Inventory; SCL-90: Symptom Checklist-90; SCQ: Social Communication Questionnaire; SF-36: 36-Item Short Form Health Survey; STAI: State-Trait Anxiety Inventory; TAI: Trait Anxiety Inventory; VABS-II: Vineland Adaptive Behavior Scales - Second Edition; VBM: Voxel-Based Morphometry; VLMT: Verbal Learning Memory Test.

*Both Schneider (42) and Yamanbaeva (54) are secondary analyses of the initial work done by Schaub (39). While all three studies reported findings from the same initial participant population (n=47), the secondary analyses had varying sample sizes, based on the indication. For this reason, sample sizes listed in this table have remained specific to each individual study.

**TABLE 2:** Risk of Bias (RoB) Assessment.

Study	Sequence Generation		Allocation Concealment		Blinding of Participants and Personnel		Blinding of Outcome Assessment		Incomplete Outcome Data	
	RoB	Comments	RoB	Comments	RoB	Comments	RoB	Comments	RoB	Comments
Rode (48)	Low	Participants were randomly assigned to treatment groups using a computerized randomization list and block randomization.	Low	Study was double-blind, all group assignment and sachet labeling was done by a university staff member not involved with the study. The study was blinded for all participants and staff.	Low	Study was double-blind, all group assignment and sachet labeling was done by a university staff member not involved with the study. The study was blinded for all participants and staff.	Low	Study was double-blind, all group assignment and sachet labeling was done by a university staff member not involved with the study. The study was blinded for all participants and staff.	Low	Subjects that were excluded were documented with reasoning.
Ascone (49)	Low	Randomization was via a list created with a random sequence generator.	Low	Assignment was done by a third person who was unrelated to the study.	Low	Assessors and participants were blind to group allocation. The randomisation list was given to the first author after data collection was finished.	Low	Assessors and participants were blind to group allocation. The randomisation list was given to the first author after data collection was finished.	Low	Subjects that were excluded were documented with reasoning.
Papalini (52)	Low	Participants were randomized using a computer-generated block randomization scheme by Winclove.	Low	No research personnel involved with participants could adjust the randomization or discern what products participants were receiving.	Low	No research personnel involved with participants could adjust the randomization or discern what products participants were receiving.	Low	No research personnel involved with participants could adjust the randomization or discern what products participants were receiving.	Low	Subjects who were excluded were documented with reasoning.
Billeci (53)	Low	Random independent block allocation using a randomized sequence with a random order of interventions.	Low	Allocation information was sealed in envelopes	Low	Subjects, caregivers, all research investigators and all outcome assessors were blinded to treatment group assignment of all subjects till the end of data collection and analysis.	Low	Subjects, caregivers, all research investigators and all outcome assessors were blinded to treatment group assignment of all subjects till the end of data collection and analysis.	Low	Subjects who were excluded were documented with reasoning.
Schaub (39)	Low	Block randomization was performed in a 1:1 ratio by an independent researcher using a computer-based randomization algorithm to avoid systematic biases	Low	States that investigators and assessors were blinded during data collection and analysis, but the details for how this was done are not stated	Low	States that investigators and assessors were blinded during data collection and analysis, but the details for how this was done are not stated	Low	States that investigators and assessors were blinded during data collection and analysis, but the details for how this was done are not stated	Low	Subjects that were excluded were documented with reasoning.
Schneider (42)	Low	Block randomization was performed in a 1:1 ratio by an independent researcher using a computer-based randomization algorithm to avoid systematic biases	Low	States that investigators and assessors were blinded during data collection and analysis, but the details for how this was done are not stated	Low	States that investigators and assessors were blinded during data collection and analysis, but the details for how this was done are not stated	Low	States that investigators and assessors were blinded during data collection and analysis, but the details for how this was done are not stated	Low	Subjects that were excluded were documented with reasoning.
Yaman-baeva (54)	Low	Block randomization was performed in a 1:1 ratio by an independent researcher using a computer-based randomization algorithm to avoid systematic biases	Low	States that investigators and assessors were blinded during data collection and analysis, but the details for how this was done are not stated	Low	States that investigators and assessors were blinded during data collection and analysis, but the details for how this was done are not stated	Low	States that investigators and assessors were blinded during data collection and analysis, but the details for how this was done are not stated	Low	Subjects that were excluded were documented with reasoning.
Pinto-Sanchez (55)	Low	Block randomization stratified by gender and IBS status using a computer program	Low	Allocation codes were sealed in opaque envelope. Treatment allocation was concealed from participants and study staff	Low	Treatment allocation was concealed from participants and study staff. Treatment products were indistinguishable in terms of package, color, taste, and consistency, and were blinded to subjects, investigators and support staff.	Low	Treatment allocation was concealed from participants and study staff. Treatment products were indistinguishable in terms of package, color, taste, and consistency, and were blinded to subjects, investigators and support staff.	Low	Subjects that were excluded were documented with reasoning.

**TABLE 3:** Risk of Bias (RoB 2) Assessment for Crossover Trials.

Study	Randomization Process		Period and Carryover Effects		Deviations from Intended Intervention		Missing Outcome Data		Measurement of the Outcome		Selection of Reported Results	
	RoB	Comments	RoB	Comments	RoB	Comments	RoB	Comments	RoB	Comments	RoB	Comments
Allen (50)	High	All participant received placebo first and then probiotic	High	Since all participant received placebo first, any placebo effect could have carried over to the probiotic period.	Some/High	There was no appropriate analysis to estimate the effect of assignment or adherence to treatment.	Low	All outcome data with adequate response or quality was reported.	Low	Though outcome assessors were aware of the intervention received, it is unlikely this could influence the assessment	Low	It is unlikely reported results were from multiple eligible outcome measures, or analyses.
Kelly (51)	Low	Used a randomized placebo-controlled cross-over repeated measures design but no mention as to how allocation was randomized.	Some	Period effects were not included in the analysis	Some/High	There was no appropriate analysis to estimate the effect of assignment or adherence to treatment.	Low	All outcome data with adequate response or quality was reported.	Low	It is unlikely outcome assessors were aware of the intervention received although it is not clearly reported. It is unlikely this could influence the assessment	Low	It is unlikely reported results were from multiple eligible outcome measures, or analyses.

### 2.4 Study quality

Quality assessment of articles was completed using Covidence’s built-in, Cochrane Handbook for Systematic Reviews of Interventions, Risk of Bias (RoB) tool. The Cochrane RoB tool assesses the risk of bias for the following domains: sequence generation, allocation concealment, blinding of participants and personnel, blinding of outcome assessment, and incomplete outcome data. Most studies presented with a low level of bias. Studies where there was no mention of blinding to participants, personnel, outcome assessors, or allocation of treatment, were assigned a “high” RoB judgment. The Revised Cochrane RoB tool (RoB 2) for crossover trials was used for the two crossover trials included in this review. A detailed summary of the quality assessment can be found in **Table 2**, **3**. Quality assessment was conducted by EF and independently verified by CS.

## 3 Results

### 3.1 Study characteristics

Of the 10 papers that passed all inclusion criteria and were included for data extraction and analysis, 5 included studies that involved healthy participants and the remaining 5 involved participants with psychiatric disorders. Though the search strategy and inclusion criteria were designed to capture studies using any microbial-based intervention, all 10 of the articles that passed inclusion criteria used probiotics containing bacteria from the *Bifidobacterium* and *Lactobacillus* genera along with various other strains and species. The neuroimaging techniques used in these studies included MRI (*n* = 2) (48, 49), fMRI (*n* = 5) (39, 42, 52, 54, 55), voxel-based morphometry (*n* = 2) (39, 48), and EEG (*n* = 3) (50, 51, 53). Healthy control studies included a total of 190 participants, with sample sizes ranging from *n* = 22 to *n* = 59 and an average sample size of 38. The psychiatric-focused studies involved 137 participants in total, including individuals with autism spectrum disorder (ASD) (*n* = 46), MDD (*n* = 47), and irritable bowel syndrome (IBS) with mild to moderate depression and/or anxiety symptoms (*n* = 44). It is important to clarify that participant totals for the MDD indication have been reported from the same patient population, as the initial work was published by Schaub (2022) and secondary analyses were later conducted by Schneider (2023) and Yamanbaeva (2023). While all three studies reported findings from the same initial participant population, the secondary analyses had varying sample sizes. To maintain accuracy and reflect this distinction, study-specific sample sizes and a detailed summary of study characteristics can be found in **Table 1**. Given that each of these studies reported unique findings, paired with the limited available neuroimaging data associated with probiotic supplementation within psychiatry, the decision was made to retain all three studies in the review. The majority of studies in this review were found to have a low risk of bias, while the two crossover studies demonstrated some significant risk of bias relating to the randomization process, potential carryover and period effects, and the lack of analyses to estimate the effects of assignment or adherence to treatment.

### 3.2 Healthy indication

In the work completed by Allen and colleagues (2016), healthy male volunteers (*n* = 22) received placebo for four weeks before receiving probiotic intervention for another four weeks. Through the use of EEG data, they found probiotic intervention to be associated with an increase in midline frontal (Fz) mobility and a decrease in midline central (Cz) theta power. These EEG changes were correlated with subtle improvements in visuospatial memory as assessed by the Cambridge Neuropsychological Test Automated Battery (50). It is possible that cognitive improvement may have been inflated due to participants seeing it twice previously, prior to the assessment after the probiotic phase, however this risk of bias is unlikely to have greatly affected the EEG measures.

Papalini and colleagues (2019) had healthy participants (*n* = 58) receive either probiotic intervention (*n* = 29) or placebo (*n* = 29) for four weeks. They reported that the probiotic intervention group displayed an increased buffer to the effects of stress on working memory performance, in relation to the placebo group. Physical stress was induced using the Social Evaluated Cold Pressor Test, where participants submerged their hand in cold water for up to 3 minutes. Psychological stress was induced by having an unknown researcher display neutral and socially distant behavior, and instructing participants to look into a camera during the cold-water test to have their facial expressions recorded. Participants underwent the digit span test to evaluate their working memory before and after the Social Evaluated Cold Pressor Test, pre- and post-intervention. Participants in the probiotic group were less negatively affected by stress, than the placebo group. This increased buffer effect was especially seen in individuals with probiotic-induced decreases in activity in the right prefrontal cortex during stress-related working memory tests (52).

Lastly, in the study conducted by Rode and colleagues (2022), healthy participants (*n* = 22) were given either a probiotic mixture or placebo for 4 weeks, followed by a 4-week washout period, and then switching to the other arm (either probiotic or placebo depending on which they started with), for an additional 4 weeks. They reported probiotic intervention to be associated with a significant increase in the functional connectivity between the default mode network and postcentral gyrus and superior parietal lobule, as well as between the language network and the middle temporal gyrus, inferior temporal gyrus, and lateral occipital cortex. Significantly reduced functional connectivity was observed after probiotic intervention between the left supramarginal gyrus (within the salience network) and the postcentral gyrus, as well as between the right supramarginal gyrus (within the salience network) and the brain stem, precuneus cortex, cerebellum, and supracalcarine cortex. Significantly reduced functional connectivity was also observed between the frontoparietal network and the middle frontal and precentral gyri (48).

In the two remaining studies, both Kelly and colleagues (2017) and Ascone and colleagues (2022) reported no significant differences between the probiotic and placebo groups after intervention (49, 51). Any associated risk of bias identified for Kelly (2017) is unlikely to have influenced these results.

### 3.3 Psychiatric indication

As previously mentioned, three of the MDD studies had reported results on the same population of participants, due to Schneider and colleagues (2023) and Yamanbaeva and colleagues (2023) reporting secondary analyses of the work by Schaub and colleagues (2022) (39, 42, 54). In the original study by Schaub and colleagues (2022), participants received either probiotic or placebo (in addition to treatment as usual) for a period of 31 days, with a follow-up assessment 4 weeks after the intervention. They found a significant increase in grey matter volume in the calcarine sulcus extending into the lingual gyrus after probiotic intervention, when compared to the placebo group. When looking at activity changes pre- and post-intervention, there was a significant decrease in right and left putamen activation during neutral face processing, in the probiotic group. There were no significant activation changes in this region, for the placebo group. For clinical results, both the probiotic group and the placebo group had a significant decrease in HAM-D scores over time, however the effect was stronger in the probiotic group (39).

A secondary analysis conducted by Schneider and colleagues (2023) found a decrease in left hippocampus activation in the probiotic group after intervention, in a working memory task. When the activity changes in the left hippocampus were correlated with reaction time during the task, it was found that decreased hippocampus activity in the probiotic group was correlated with a decreased reaction time during the task. In contrast, increased hippocampus activity in the placebo group was correlated with a decreased reaction time during the task (42).

Another secondary analysis conducted by Yamanbaeva and colleagues (2023) found the probiotic group to have significantly higher fractional anisotropy in the uncinate fasciculus, as compared to placebo. The probiotic group also maintained a stable mean diffusivity during and after treatment, whereas the placebo group had a significant increase in mean diffusivity post-treatment. When examining resting state functional connectivity (rsFC) in the probiotic group, increased connectivity was observed between the subcallosal cortex, left temporal pole, right and left hippocampus, and right and left amygdala, to a cluster in the precuneus. Increased connectivity was also observed between the left orbitofrontal cortex and a cluster in the left superior parietal lobule extending to the left posterior supramarginal gyrus. Decreased connectivity was observed between the subcallosal cortex, left hippocampus, and right amygdala, with this cluster. When looking at blood perfusion, they reported the mean cerebral blood flow in the hippocampus to be significantly higher in the placebo group than the probiotic group (54).

A study by Pinot-Sanchez and colleagues (2017) examined the use of a probiotic product in a population with IBS and diarrhea or a mixed-stool pattern, and mild to moderate anxiety and/or depression (*n* = 44). Participants were randomized to receive either probiotic or placebo for a period of six weeks, with fMRI data collected both before and after treatment. Post-intervention, the probiotic group displayed reduced activation of the amygdala and frontal and temporal cortices, and increased activation of occipital regions in response to fear stimuli, when compared to the placebo group. Within the probiotic group, reduced amygdala activation was significantly correlated with decreased depression scores and was more likely to occur in patients with adequate relief of IBS symptoms, than those without it (55).

Lastly, Billeci and colleagues (2023) investigated the use of a probiotic mixture in children aged 18–72 months with ASD (*n* = 46). They were given either probiotic or placebo for 6 months, with EEG measures conducted pre- and post-treatment for changes in power, coherence, and asymmetry. In the probiotic group, both beta and gamma bands displayed a decrease in power in the right and left frontopolar regions and an increase in that frontopolar coherence. The probiotic group also displayed a decrease in frontal asymmetry in delta band, while the placebo group displayed an increase in frontopolar asymmetry in alpha band. Lower raw field potential power in gamma band was found to be correlated with a lower number of caregiver-reported repetitive behaviors (e.g., stereotyped and/or self-injurious behavior, etc.), while higher frontopolar coherence in beta and gamma bands was correlated with higher writing skill scores on a semi-structured interview with caregivers. In addition to this, children with lower levels of tumor necrosis factor alpha (TNF-α) post-intervention also showed higher frontopolar coherence in gamma band.

## 4 Discussion

### 4.1 Main findings

Ten studies investigating the effects of microbial interventions on neurobiological structures and functions met the criteria for inclusion in this systematic review. All studies used probiotic formulations containing primarily bacteria from the *Lactobacillus* and *Bifidobacterium* genera. No studies using non-probiotic microbial interventions, such as FMT and prebiotics, were identified, indicating a clear and significant gap in the literature when it comes to neurobiological changes associated with these treatments in the context of psychiatry. Of the five studies conducted with healthy populations, three found significant neurobiological changes after probiotic intervention (48, 50, 52), specifically within domains such as the default mode network, working memory performance areas, and brain activity measured by EEG’s. While these changes maintain relevance within the field of psychiatry, there were no significant differences in psychiatric symptoms between the probiotic and placebo groups. The remaining two healthy indication studies found no significant differences between the probiotic and placebo groups (49, 51). As for the five studies conducted with psychiatric populations, all found significant neurobiological changes resulting from probiotic interventions that were either in the direction of a healthier profile or correlated with improved psychiatric symptoms (39, 42, 53–55).

While two studies with healthy populations did not report any significant changes between probiotic and placebo groups (49, 51), other work reported quite the opposite. Rode (2022) found probiotic intervention in healthy individuals to be associated with decreased grey matter volume in the left supramarginal gyrus, decreased functional connectivity between both supramarginal gyri, and multiple other brain regions associated with emotional regulation, the salience network, and the default mode network (48). They postulate that the decrease in grey matter and functional connectivity could indicate higher brain efficiency. Interestingly, other work has found that increased grey matter in the inferior parietal lobule, which contains the supramarginal gyrus, has been observed in first episode treatment-naïve individuals with schizophrenia, when compared to healthy controls (56). Alterations in the default mode and salience networks have also been observed in many other mood and anxiety-related disorders (57, 58), suggesting the findings by Rode (2022) to have clear relevance in the context of psychiatric illnesses and disorders (48).

If the focus shifts to cognition, the studies conducted by Allen and colleagues (2016) as well as Papalini and colleagues (2019) both offer intriguing insights. Allen and colleagues (2016) observed an increase in EEG Fz mobility, a marker of prefrontal cortex (PFC) activity, and a reduction in Cz theta power, correlating with improved memory performance after probiotic intervention (50). Similarly, Papalini and colleagues (2019) found that probiotics provided an increased resilience against stress-related challenges in working memory, specifically for those showing decreased activity in the right prefrontal cortex during cognitive control tasks (52). Both findings are relevant within a psychiatric context, as decreased prefrontal cortex activity and compromised working memory have both been observed in other disorders such as posttraumatic stress disorder (PTSD), bipolar disorder (59), manic symptoms (60), and schizophrenia (61, 62). Given that the PFC is crucial for its involvement in executive functioning and emotional regulation, it is without surprise that PFC activity is frequently hypoactive both at rest (62) and under stress (63), in psychiatric populations. Utilizing microbial treatment strategies such as probiotics, may aid in optimizing PFC functioning and serve as a tool to address any cognitive and/or emotional deficits associated with these disorders.

In psychiatric populations, probiotics continue to appear to have promising effects. In MDD, the three analyses of the cohort from the Schaub (2022) trial found various significant structural and functional differences associated with probiotic intervention, relating primarily to the limbic system both at rest and during tasks. These differences included increased grey matter in the calcarine sulcus, decreased putamen and hippocampus activation during neutral face processing and working memory tasks respectively, increased fractional anisotropy in the uncinate fasciculus and maintenance of mean diffusivity, and changed rsFC between precuneus and superior parietal lobule and various limbic structures (39, 42, 54).

Previous work has documented that individuals with depression can exhibit hyperactivity in structures associated with the limbic system, such as the putamen region and amygdala (64). Given these structures’ involvement with facial expression recognition (65), it is postulated that hyperactivity in this area could contribute to a tendency to interpret neutral faces, as more negative and/or threatening (66, 67). Interestingly, Schaub and colleagues (2022) reported a decrease in putamen activation following probiotic intervention, while viewing neutral faces in a population with depression. The authors suggested these findings could be indicative of individuals experiencing a more balanced emotional response, interpreting neutral faces less negatively, or reaction less intensely to negative stimuli. Though these conclusions cannot be certain, the overall decrease in putamen activation serves as a marker for identifying neurobiological changes associated with both the disorder and response to probiotics. Similarly, Schneider and colleagues (2023) reported reduced hippocampus activation during working memory tasks in the same population, after probiotic intervention, whereas the placebo group had an increase in hippocampal activation (42). This is aligned with other work done in the field, as the hippocampal region has been noted to be hyperactive during rest in a population with depression (68). While most of hippocampal work has been done through a structural lens, such as identifying volumetric changes and correlating it to clinical outcomes (69), structural data collection and analysis is beginning to catch up. Smith and colleagues (2017) also reported similar findings with a working memory task, where vortioxetine produced a reduction in hippocampal activity for both individuals with MDD and health controls, as compared to placebo (70). Yamanbaeva and colleagues (2023) provided further insights into the effects of probiotics on brain function in MDD, by observing a stabilizing effect of mean diffusivity in the uncinate fasciculus for participants who received probiotics, whereas the placebo group experienced an increase. They also reported changes in rsFC between limbic structures and other brain regions, with an increase in connections to the precuneus and decrease in connections to the left superior parietal lobule (54).

Pinto-Sanchez and colleagues (2017) examined the use of probiotics in a population with IBS. They noted reduced neural responses to negative stimuli in the amygdala and fronto-limbic system, which both play essential roles in the brains’ emotional regulation, memory, and decision making (55). Furthermore, Billeci and colleagues (2023) documented EEG changes towards a neuro-typical profile in preschoolers with ASD, post-probiotic intervention. These changes included decreased power in frontopolar regions and increased coherence in beta and gamma bands, which may reflect an improvement and restoration in the imbalance between excitatory and inhibitory neurons, as well as a change in brain connectivity towards a typical pattern (53).

In general, many of the studies identified in this review found changes associated with probiotic use in brain regions and networks related to the limbic system (42, 48, 54, 55), prefrontal cortex (48, 53, 55), and striatum (39, 52). These brain regions have strong psychiatric implications, particularly when it comes to mood and anxiety-related symptoms and disorders. These regions are heavily involved with emotion regulation, recognition, and functional networks such as the default mode network. Activity in these regions and functional networks have been consistently found to be disrupted in mood disorders such as major depressive disorder (71–73), with the default mode network being linked to key symptoms like rumination (74, 75). The consistent shift towards a healthier profile observed in both healthy and psychiatric populations suggests that probiotics with bacterial species primarily of the *lactobacillus* and *bifidobacterium* genera, may have a transdiagnostic effect on areas of the brain associated with symptoms of mood disorders.

The primary mechanisms by which the gut microbiome is able to enact change on the central nervous system are thought to be through interactions with the immune system, HPA-axis, and vagus nerve. Interactions with these systems may elicit changes in brain regions and networks that were observed in the studies included in this review, such as the finding from Allen (2016) which reported a significant reduction in total cortisol output in the probiotic group when compared to the placebo group. It has been proposed that the gut microbiome can influence the immune system and inflammation through a variety of ways, including reducing the permeability of the intestinal endothelium to in turn reduce the inflammatory response driven by pathogens translocating from the intestinal lumen into the body (76), and producing metabolites like short-chain fatty acids that have inherent anti-inflammatory properties (77). Systemic inflammation, and in turn neuroinflammation, has been associated with a variety of changes in the structure and function of limbic and prefrontal regions (78, 79), and could thus be a potential mechanism for the changes observed in some of these studies. Though some studies that assessed for inflammatory markers found no significant differences between groups, it is possible that changes in immune functioning played a role in eliciting the findings from the other studies. Similarly, regulation of the HPA-axis through probiotic use could explain some of the findings as HPA-axis activity has been associated with changes in amygdala, hippocampus, and prefrontal cortex activity, but two of the three studies that assessed cortisol levels, a marker for HPA-axis activity, found no differences between groups. Allen (2016) was the only study to find a significant reduction in total cortisol output in the probiotic group compared to the placebo group, but HPA-axis involvement still remains a possibility for the studies that did not collect cortisol or other markers of HPA-axis activity.

When it comes to interactions with the vagus nerve and vagal tone, preclinical models have found evidence to suggest long- and short-chain fatty acids produced by the gut microbiome can indirectly and directly stimulate vagal afferent fibers respectively (80). Vagal activity can also be affected by the gut microbiome indirectly through interactions with enteroendocrine cells and the HPA-axis (81). It is possible that changes in vagus nerve activity and tone could have driven the neurobiological changes observed in the studies included in this review, as vagus nerve stimulation has been associated with changes in prefrontal cortex activity and monoamine concentrations in the brain that would in turn effect the activity of various limbic and striatal regions (21). It is unknown how significantly the probiotic administration affected the vagus nerve in the aforementioned studies as they did not include any measures of vagus nerve activity, such as heart rate variability, but the lack of significant changes observed in inflammatory and HPA-axis markers suggests the vagus nerve may have been a significant mechanism of action for the observed neurobiological changes associated with probiotic use.

### 4.2 Limitations

Some key limitations should be considered when interpreting the findings of this review. First, despite search terms including a variety of microbial interventions, all included papers maintained the use of probiotic intervention rather than a multitude of different interventions. There is also a clear lack of literature assessing such changes associated with probiotic administration in psychiatric populations, as only five studies from 3 distinct cohorts identified in this review maintained a primary psychiatric scope. While this was anticipated due to the novelty of using microbial treatments for use within psychiatry, it is impossible to draw any firm conclusions on the influence of probiotics, let alone other microbial interventions, on specific disorders such as depression. The psychiatric-indication studies also had relatively small sample sizes which can be common for study designs involving frequent and/or numerous study assessments (e.g., neuroimaging, clinical scales, etc.), due to the increased burden on participants and associated inconsistence with compliance. Furthermore, most studies maintained a relatively short intervention period and lacked in-depth, long-term, evaluations after probiotic treatment. A common follow-up timepoint is often set at 4-weeks post-treatment, however that is hardly sufficient to observe any long-term significant changes in brain structure and function. While some findings demonstrated a positive effect on psychological symptoms and cognition following probiotic treatment, the extent of that sustained improvement requires further investigation.

In the Schaub and colleagues (2022) study, they combined probiotic supplementation with standard treatment as usual for depression. While using probiotics as an add-on treatment is another necessary step towards understanding how microbial treatments influence mood, it also poses as a limitation when attempting to uncover any associated neural effects of probiotics. It raises the question as to whether any observed changes are in response to solely probiotic administration, antidepressant administration, or a combination of both treatments. This interplay between concurrent probiotic and antidepressant use needs further consideration, as some antidepressants have been found to have their own independent effects on the gut microbiome (82). Lastly, each study included in this review used different probiotic interventions. Though most probiotics used in these studies contained bacteria from the *Lactobacillus* and *Bifidobacterium* genera, the specific species, strains, doses, and mix of bacteria differed significantly between each probiotic intervention. This makes it difficult to directly compare the effects of probiotics and inherently limits the generalizability of the findings.

### 4.3 Future directions

This systematic review provides an overview of the current structural and functional changes correlated with probiotic treatment in healthy and psychiatric populations shown by various neuroimaging techniques. Most studies demonstrated that probiotic intervention was correlated with changes in brain structures, networks, or functioning that have been associated with various psychiatric disorders (57, 58, 68). However, these results are still far and few. Further integration of neuroimaging use within double-blind randomized controlled trials with large and diverse sample sizes, along with in-depth follow-up periods, is necessary to establish the relationships between probiotic treatment, psychiatric symptoms, and neurobiological changes and/or biomarkers. Additionally, any neuroimaging investigations using non-probiotic interventions would be crucial in determining the generalizability of these findings and aid in identifying any unique neurobiological characteristics associated with microbial interventions.

In terms of precision medicine, establishing biomarkers and incorporating their use into clinical psychiatric practice can aid in confirming diagnoses, predicting treatment outcomes, and better understanding the prognosis of disorders. Utilizing personalized approaches to patient care goes beyond the current status of their psychiatric state, as it integrates the unique variability of each individuals’ genetic makeup, environmental factors, and lifestyle differences (83). Such information is invaluable for the continued development and improvement of effective treatment guidelines and medications within psychiatry. Given the novelty of using microbial therapeutics for mood-related changes and disturbances, neuroimaging serves as a potential tool to understand how gut-focused treatments can influence brain function and behavior.

## Data Availability

The original contributions presented in the study are included in the article/**Supplementary Material**. Further inquiries can be directed to the corresponding author.

## Glossary

ADHD: Attention Deficit Hyperactivity Disorder
ADOS-2: Autism Diagnostic Observation Schedule – Second Version
ASD: Autism Spectrum Disorder
BDI: Beck Depression Inventory
BDI-II-R: Beck Depression Inventory (2e) – Revised
BSI: Brief Symptom Inventory
CARS: Childhood Autism Rating Scale
CBCL: Child Behavior Checklist
CD: Clostridium Difficile
CDI: McArthur-Bates Communicative Development Inventories
CFU: Colony Forming Unit
CPSS: Cohen Perceived Stress Scale
CT: Computerized Tomography
Cz: Midline Central
DBRCT: Double-Blind Randomized Controlled Trial
DOW: Diary of Workload
DTI: Diffusion Tensor Imaging
EEG: Electroencephalography
EQ-5D-5L: EuroQol 5-Dimension 5-Level Health-Related Quality of Life Index
FMT: Fecal Microbiota sTransplantation
fMRI: functional Magnetic Resonance Imaging
fNIRS: functional Near-Infrared Spectroscopy
Fz: Midline Frontal
GBA: Gut-Brain Axis
GI: Gastrointestinal
GMDS-R: Griffiths Mental Development Scales - Extended Revised
GSI: Gastrointestinal Severity Index
HAD: Hospital Anxiety and Depression Scale
HAM-D: Hamilton Depression Rating Scale
HPA: Hypothalamic-Pituitary-Adrenal
IBD: Irritable Bowel Disease
IBS: Irritable Bowel Syndrome
KSD: Karolinska Sleep Diary
LEIDS-R: Leiden Index of Depression Sensitivity - Revised
MDD: Major Depressive Disorder
MRI: Magnetic Resonance Imaging
OCD: Obsessive Compulsive Disorder
PET: Positron Emission Tomography
PFC: Prefrontal Cortex
PHQ-15: Patient Health Questionnaire-15
PRISMA: Preferred Reporting Items for Systematic Reviews and Meta-Analyses
PSS: Perceived Stress Scale
PTSD: Posttraumatic Stress Disorder
QoL: Quality of Life
RBS-R: Repetitive Behavior Scale - Revised
RCT: Randomized Controlled Trial
RoB: Risk of Bias
RoB 2: Revised Cochrane RoB Tool
rsFC: Resting State Functional Connectivity
RSQ: Response Styles Questionnaire
SAI: State Anxiety Inventory
SCL-90: Symptom Checklist-90
SCQ: Social Communication Questionnaire
SF-36: 36-Item Short Form Health Survey
STAI: State-Trait Anxiety Inventory
TAI: Trait Anxiety Inventory
TNF-α: Tumor Necrosis Factor Alpha
UC: Ulcerative Colitis
VABS-II: Vineland Adaptive Behavior Scales – Second Edition
VBM: Voxel-Based Morphometry
VLMT: Verbal Learning Memory Test
