# Supplementary material for: Neurobiological effects of microbial treatments within psychiatry: a systematic review
Source: Front Psychiatry. 2026 Apr 20;17:1745964. doi: 10.3389/fpsyt.2026.1745964 (PMC13137161; doi:10.3389/fpsyt.2026.1745964)
Supplement: Supplementary file 1 [file SupplementaryFile1.docx]

**TABLE 4. Search Strategy**

| **#** | **Search Term** |
| --- | --- |
| **Database: MEDLINE, PsycINFO, EMBASE via Ovid** | |
| 1. | Magnetic resonance imaging.mp. [mp=ti, bt, ab, ot, nm, hw, fx, kf, ox, px, rx, ui, an, sy, ux, mx, tn, dm, mf, dv, dq, tc, id, tm] |
| 2. | MRI.mp. [mp=ti, bt, ab, ot, nm, hw, fx, kf, ox, px, rx, ui, an, sy, ux, mx, tn, dm, mf, dv, dq, tc, id, tm] |
| 3. | Functional magnetic resonance imaging.mp. [mp=ti, bt, ab, ot, nm, hw, fx, kf, ox, px, rx, ui, an, sy, ux, mx, tn, dm, mf, dv, dq, tc, id, tm] |
| 4. | fMRI.mp. [mp=ti, bt, ab, ot, nm, hw, fx, kf, ox, px, rx, ui, an, sy, ux, mx, tn, dm, mf, dv, dq, tc, id, tm] |
| 5. | Diffusion tensor imaging.mp. [mp=ti, bt, ab, ot, nm, hw, fx, kf, ox, px, rx, ui, an, sy, ux, mx, tn, dm, mf, dv, dq, tc, id, tm] |
| 6. | DTI.mp. [mp=ti, bt, ab, ot, nm, hw, fx, kf, ox, px, rx, ui, an, sy, ux, mx, tn, dm, mf, dv, dq, tc, id, tm] |
| 7. | positron emission tomography.mp. [mp=ti, bt, ab, ot, nm, hw, fx, kf, ox, px, rx, ui, an, sy, ux, mx, tn, dm, mf, dv, dq, tc, id, tm] |
| 8. | Electroencephalography.mp. [mp=ti, bt, ab, ot, nm, hw, fx, kf, ox, px, rx, ui, an, sy, ux, mx, tn, dm, mf, dv, dq, tc, id, tm] |
| 9. | EEG.mp. [mp=ti, bt, ab, ot, nm, hw, fx, kf, ox, px, rx, ui, an, sy, ux, mx, tn, dm, mf, dv, dq, tc, id, tm] |
| 10. | Computerized tomography.mp. [mp=ti, bt, ab, ot, nm, hw, fx, kf, ox, px, rx, ui, an, sy, ux, mx, tn, dm, mf, dv, dq, tc, id, tm] |
| 11. | CT.mp. [mp=ti, bt, ab, ot, nm, hw, fx, kf, ox, px, rx, ui, an, sy, ux, mx, tn, dm, mf, dv, dq, tc, id, tm] |
| 12. | Functional near-infrared spectroscopy.mp. [mp=ti, bt, ab, ot, nm, hw, fx, kf, ox, px, rx, ui, an, sy, ux, mx, tn, dm, mf, dv, dq, tc, id, tm] |
| 13. | fNIRS.mp. [mp=ti, bt, ab, ot, nm, hw, fx, kf, ox, px, rx, ui, an, sy, ux, mx, tn, dm, mf, dv, dq, tc, id, tm] |
| 14. | ultrasound.mp. [mp=ti, bt, ab, ot, nm, hw, fx, kf, ox, px, rx, ui, an, sy, ux, mx, tn, dm, mf, dv, dq, tc, id, tm] |
| 15. | magnetoencephalography.mp. [mp=ti, bt, ab, ot, nm, hw, fx, kf, ox, px, rx, ui, an, sy, ux, mx, tn, dm, mf, dv, dq, tc, id, tm] |
| 16. | probiotic*.mp. [mp=ti, bt, ab, ot, nm, hw, fx, kf, ox, px, rx, ui, an, sy, ux, mx, tn, dm, mf, dv, dq, tc, id, tm] |
| 17. | prebiotic*.mp. [mp=ti, bt, ab, ot, nm, hw, fx, kf, ox, px, rx, ui, an, sy, ux, mx, tn, dm, mf, dv, dq, tc, id, tm] |
| 18. | postbiotic*.mp. [mp=ti, bt, ab, ot, nm, hw, fx, kf, ox, px, rx, ui, an, sy, ux, mx, tn, dm, mf, dv, dq, tc, id, tm] |
| 19. | psychobiotic*.mp. [mp=ti, bt, ab, ot, nm, hw, fx, kf, ox, px, rx, ui, an, sy, ux, mx, tn, dm, mf, dv, dq, tc, id, tm] |
| 20. | fecal microbiota transplant*.mp. [mp=ti, bt, ab, ot, nm, hw, fx, kf, ox, px, rx, ui, an, sy, ux, mx, tn, dm, mf, dv, dq, tc, id, tm] |
| 21. | FMT.mp. [mp=ti, bt, ab, ot, nm, hw, fx, kf, ox, px, rx, ui, an, sy, ux, mx, tn, dm, mf, dv, dq, tc, id, tm] |
| 22. | stool transplant.mp. [mp=ti, bt, ab, ot, nm, hw, fx, kf, ox, px, rx, ui, an, sy, ux, mx, tn, dm, mf, dv, dq, tc, id, tm] |
| 23. | bacteriotherapy.mp. [mp=ti, bt, ab, ot, nm, hw, fx, kf, ox, px, rx, ui, an, sy, ux, mx, tn, dm, mf, dv, dq, tc, id, tm] |
| 24. | microbe therapy.mp. [mp=ti, bt, ab, ot, nm, hw, fx, kf, ox, px, rx, ui, an, sy, ux, mx, tn, dm, mf, dv, dq, tc, id, tm] |
| 25. | microbe transfer.mp. [mp=ti, bt, ab, ot, nm, hw, fx, kf, ox, px, rx, ui, an, sy, ux, mx, tn, dm, mf, dv, dq, tc, id, tm] |
| 26. | depression.mp. [mp=ti, bt, ab, ot, nm, hw, fx, kf, ox, px, rx, ui, an, sy, ux, mx, tn, dm, mf, dv, dq, tc, id, tm] |
| 27. | depress*.mp. [mp=ti, bt, ab, ot, nm, hw, fx, kf, ox, px, rx, ui, an, sy, ux, mx, tn, dm, mf, dv, dq, tc, id, tm] |
| 28. | MDD.mp. [mp=ti, bt, ab, ot, nm, hw, fx, kf, ox, px, rx, ui, an, sy, ux, mx, tn, dm, mf, dv, dq, tc, id, tm] |
| 29. | bipolar*.mp. [mp=ti, bt, ab, ot, nm, hw, fx, kf, ox, px, rx, ui, an, sy, ux, mx, tn, dm, mf, dv, dq, tc, id, tm] |
| 30. | bipolar disorder.mp. [mp=ti, bt, ab, ot, nm, hw, fx, kf, ox, px, rx, ui, an, sy, ux, mx, tn, dm, mf, dv, dq, tc, id, tm] |
| 31. | adhd*.mp. [mp=ti, bt, ab, ot, nm, hw, fx, kf, ox, px, rx, ui, an, sy, ux, mx, tn, dm, mf, dv, dq, tc, id, tm] |
| 32. | attention deficit hyperactivity disorder.mp. [mp=ti, bt, ab, ot, nm, hw, fx, kf, ox, px, rx, ui, an, sy, ux, mx, tn, dm, mf, dv, dq, tc, id, tm] |
| 33. | autism*.mp. [mp=ti, bt, ab, ot, nm, hw, fx, kf, ox, px, rx, ui, an, sy, ux, mx, tn, dm, mf, dv, dq, tc, id, tm] |
| 34. | ASD.mp. [mp=ti, bt, ab, ot, nm, hw, fx, kf, ox, px, rx, ui, an, sy, ux, mx, tn, dm, mf, dv, dq, tc, id, tm] |
| 35. | anxiety*.mp. [mp=ti, bt, ab, ot, nm, hw, fx, kf, ox, px, rx, ui, an, sy, ux, mx, tn, dm, mf, dv, dq, tc, id, tm] |
| 36. | mania.mp. [mp=ti, bt, ab, ot, nm, hw, fx, kf, ox, px, rx, ui, an, sy, ux, mx, tn, dm, mf, dv, dq, tc, id, tm] |
| 37. | schizo*.mp. [mp=ti, bt, ab, ot, nm, hw, fx, kf, ox, px, rx, ui, an, sy, ux, mx, tn, dm, mf, dv, dq, tc, id, tm] |
| 38. | obsessive compulsive disorder.mp. [mp=ti, bt, ab, ot, nm, hw, fx, kf, ox, px, rx, ui, an, sy, ux, mx, tn, dm, mf, dv, dq, tc, id, tm] |
| 39. | OCD.mp. [mp=ti, bt, ab, ot, nm, hw, fx, kf, ox, px, rx, ui, an, sy, ux, mx, tn, dm, mf, dv, dq, tc, id, tm] |
| 40. | posttraumatic stress disorder.mp. [mp=ti, bt, ab, ot, nm, hw, fx, kf, ox, px, rx, ui, an, sy, ux, mx, tn, dm, mf, dv, dq, tc, id, tm] |
| 41. | PTSD.mp. [mp=ti, bt, ab, ot, nm, hw, fx, kf, ox, px, rx, ui, an, sy, ux, mx, tn, dm, mf, dv, dq, tc, id, tm] |
| 42. | 1 or 2 or 3 or 4 or 5 or 6 or 7 or 8 or 9 or 10 or 11 or 12 or 13 or 14 or 15 |
| 43. | 16 or 17 or 18 or 19 or 20 or 21 or 22 or 23 or 24 or 25 |
| 44. | 26 or 27 or 28 or 29 or 30 or 31 or 32 or 33 or 34 or 35 or 36 or 37 or 38 or 39 or 40 or 41 |
| 45. | 42 and 43 and 44 |
| **Database: Web of Science** | |
| 1 | (((((((((((((((ALL=(Magnetic resonance imaging)) OR ALL=(mri)) OR ALL=(Functional magnetic resonance imaging)) OR ALL=(fmri)) OR ALL=(diffusion tensor imaging)) OR ALL=(dti)) OR ALL=(Electroencephalography)) OR ALL=(eeg)) OR ALL=(positron emission tomography)) OR ALL=(Computerized tomography)) OR ALL=(CT)) OR ALL=(Functional near-infrared spectroscopy)) OR ALL=(fNIRS)) OR ALL=(ultrasound)) OR ALL=(magnetoencephalography) |
| 2 | ((((((((((ALL=(probiotic)) OR ALL=(fecal microbiota transplant)) OR ALL=(fecal microbiota transplantation)) OR ALL=(fmt)) OR ALL=(stool transplant)) OR ALL=(bacteriotherapy)) OR ALL=(microbe therapy)) OR ALL=(microbe transfer)) OR ALL=(prebiotic)) OR ALL=(postbiotic)) OR ALL=(psychobiotic) |
| 3 | ((((((((((((((((ALL=(depression)) OR ALL=(depressive disorder)) OR ALL=(MDD)) OR ALL=(bipolar)) OR ALL=(bipolar disorder)) OR ALL=(Attention deficit hyperactivity disorder)) OR ALL=(adhd)) OR ALL=(autism)) OR ALL=(ASD)) OR ALL=(anxiety)) OR ALL=(manic)) OR ALL=(mania)) OR ALL=(schizo)) OR ALL=(obsessive compulsive disorder)) OR ALL=(OCD)) OR ALL=(Posttraumatic stress disorder)) OR ALL=(PTSD) |
| 4 | 1 and 2 and 3 |
